# Chancroids of the Female Generative Organs

**Published:** 1893-12

**Authors:** E. C. Davis

**Affiliations:** Atlanta, Ga.; Assistant to Chair Gynecology, Atlanta Polyclinic


### CHANCROIDS OF THE FEMALE GENERATIVE ORGANS.*

By E. C. DAVIS, A. B., M. D.,
ATLANTA, GA.
Assistant to Chair Gynecology, Atlanta Polyclinic.

At all previous meetings of this Society we have listened to papers on purely obstetrical subjects. To-night I wish to direct your attention to a subject which, with a strict conformity to a proper distribution of diseases to the different branches of medicine, belongs to the domain of surgery rather than gynecology; but when I confine my remarks to the disease involving the female generative organs, then it falls under the province of gynecology, and in this category do I hope to deal with it in this paper. I trust in offering such a subject I will at least provoke a liberal discussion, which, with reference to the sex, has attracted but little attention. This fact may be explained by letters which I have received from several very distinguished men, who claim that they now meet with very few cases of this kind. The following quotation may aid somewhat in the further explanation: "Nurtured in filth, fostered by uncleanliness, thou art truly a despicable sojourner."

*Read before Atlanta Obstetrical Society, October 12, 1893.

Until 1852 all ulcers of the genitals were considered to be manifestations of the presence of syphilis, though certain men of distinction recognized the differences in induration in the different forms of ulcers, and that some were followed by corporal evidences of constitutional disease while others were not, but they were all classed under the general head, primary syphilis.

In 1852, Bassereau, a student of the learned Ricord, published a treatise, in which he openly expressed his belief in the existence of two forms of venereal ulcers. He it was who first formulated the following proposition, viz.: "No chancre which is followed by constitutional symptoms gives rise to a purely local sore, nor does a local chancre by contagion communicate an ulcer which is capable of producing syphilis."

M. Clerc, another student of Ricord, carried out this idea still further, and to him belongs the credit or, rather discredit, of attaching to the non-indurated strictly local ulcer the unfortunate appellation, chancroid, which it has been unable to discard. He gave it this name thinking it bore the same relation to cancer that varioloid bears to variola.

This opened up the contention between the two factions, one adhering to the unity, while the other attempted to overcome all opposition to the dualism of the two forms of ulcers. Not without bitter opposition was the dualism of the diseases generally established and the attention of the profession directed to a still further differentiation of this from other forms of venereal diseases.

That the impregnable bulwark, auto-inoculation, on which many so firmly relied, has tottered a little under the experiments of recent investigations, has had a tendency to revive some of the old feeling, and suggest the origination *de novo* of this trouble.

Chancroid is a local specific contagious venereal ulcer, communicated from individual to individual usually by the sexual act, but can be communicated by contact of any sort with chancroidal pus. It is *strictly* local in character and accompanied by no syphilitic manifestations.

Keyes says in his work on Genito-Urinary Diseases that "no amount of sexual excess, no degree of uncleanliness, no irritation, traumatic or chemical, however prolonged, no simple or poisonous ulceration from other specific sources (syphilis, cancer, glanders, etc.), nothing, in short, can produce chancroid except chancroid." This expresses very forcibly the belief in the dependence upon a specific micro-organism of chancroid for its manifestations. This micro-organism has not been as yet isolated with certainty, although several distinguished bacteriologists claim to have isolated it, and are at present attempting to establish its identity. This we know: that it finds its most favorable place for growth and development in filth and uncleanliness; hence, among the lowest classes, those frequenting the clinics of our colleges, it is not of such rare occurrence. The filth *per se* does not act as an exciting cause in the production of the chancroid, but it furnishes a most excellent pabulum for the growth and development of these germs when they find lodgment. These people are particularly careless about the *hygiene* of their genitals, as any one having had much experience with them in the various clinics can testify.

Chancroid seems to show a predilection as to location. Out of 387 tabulated cases which I could find recorded, almost two-thirds were situated on the external genitals. The vagina and cervix are occasionally infected, but so far I have been unable to find any authenticated account of this disease directly involving the endometrium, although I can conceive of no valid reason why they should consider this hallowed ground, knowing as well as we do how frequently gonorrhea expends its damaging force in this region. It is possible that this disease does invade the uterus, and we so far have simply failed to recognize it.

The different forms of chancroid are simple chancroid, phagedenic chancroid and mixed chancre or chancroidal chancre.

As long as the ulcer is confined to the external genitals it possesses no interest peculiar to itself or that may not be observed on the male, but when it invades the vagina and cervix, then a condition of affairs exists with peculiar interest, and resembling in some respects the intraurethral chancroid of the male.

The simple chancroid of the vagina and external genitals has to be distinguished from ulcerated abrasions, herpetic and eczematous ulcers, chancre and mucous patches. (See Genito-Urinary

Diseases with Syphilis, by E. L. Keyes, page 526.) In ulcerated abrasions the rapidity with which they respond to treatment, the superficiality of the ulcer, the absence of undermined edges, and the absence of a suspicious intercourse all tend to exclude this from chancroid. Eczema and herpes, with the exception of the rapid response to treatment, resemble the above. Herpes begins as a vesicle and follows the distribution of the nerve fibers, and is characterized by a peculiar burning pain. Chancre in vagina rarely found, as it is so small and concealed in vaginal rugæ, usually small, single, with marked induration. (The induration around the margin of chancroid, which is made up of coagulated lymph, must not be confounded with the induration of chancre.) Autoinoculation with the production of characteristic chancroid settles the question.

*Phagedenic Chancroid.*—This form of chancroid is usually found on patients who are debilitated, or have the so-called strumous diathesis and are incapable of vigorously resisting the invasion of disease. This form may also result from a careless or improper method of treatment. Sometimes where treatment is postponed through false modesty, as in the case occurring in the practice of Dr. Bransford Lewis, of St. Louis, a photograph of which he kindly sent me. This was taken of a somewhat respectable married woman, who became infected by her husband, and applied for treatment only after the disease had made the progress shown in photograph. (She made a good recovery under the use of hot antiseptic douches followed by dry dressings.)

The phagedenic form has to be differentiated from syphilitic ulcer, epithelioma and lupus. The syphilitic ulcer is almost always preceded by previous manifestations of syphilis, alopecia, rash, sore throat and other troubles with which you are perfectly familiar.

Most all previously diagnosticated cases of lupus of the genitals where the microscope has not been called into skillful requisition were not cases of lupus, but epithelioma. I have seen one case of lupus of the vulva occurring in a mulatto girl about twenty-five years of age, who was also suffering from pulmonary phthisis. Whenever lupus is suspected then should we submit secretions from the ulcer to a careful microscopic examination to detect the presence of the tubercle bacillus.

Occasionally it becomes necessary to differentiate the phagedenic chancroids from the ulcerated form of epithelioma. This is a very important distinction to make, for the treatment in the two forms of disease is radically different, and in both an early diagnosis is of vital importance, and renders the prognosis much more favorable than a later. There are a few clinical facts which will aid materially in making this distinction :

| EPITHELIOMA. | CHANCROID. |
|---|---|
| Age.—Usually occurs later in life, after thirty-five years. There are recorded cases occurring at eighteen years of age. These are rare. | Occurs usually early, but may be observed in the old. |
| Heredity.—Usually history of malignant disease of ancestors. | Plays no part. |
| Location.—When confined to cervix most frequently found on a previous laceration. | On lower fourth of vagina and sometimes on cervix. |
| Frequency.—Not of rare occurrence. Married women and those having borne children suffer oftenest. | Rare. Prostitutes, or married women who become infected by husband. |
| Development.—Usually slow at first. Begins as a hard elevation or nodule. | Rapid. Begins as a pustule, rapidly becoming an ulcer. |
| Number.—At first sinple ulcer until glandular tissue breaks down, forming another. | May be single at first, but rapidly becomes multiple. |
| Auto-inoculation. — Questionable. | Auto-inoculable. Producing characteristic chancroid. |
| Color.—Dirty, with livid edges covered over with broken down tissue. Discharging a fetid ichorous fluid very irritating. | Yellow, tawny and discharging a yelllow pus. |
| Hydrorrhea.—Hemorrhage. | No hydrorrhea; little hemorrhage. |
| No tendency to cicatrization. | Evidences of cicatrization. |
| Extends in direction of vagina and body of uterus. | On vaginal and cervical surface. |
| Buboes.—Late. Multiple enlargement of glands. | Occur early and suppurate as a rule. Usually single. |
| Microscope.—Shows presence of epitheleal scales in so-called nests. | Absence of this. |
| Cachexia.—Marked late in disease. | Usually absent. |

As long as the exciting causes of these two forms of disease are not thoroughly understood, just so long will we at times be unable to make an absolutely accurate early diagnosis and have to rely on the clinical history, together with the effect of treatment, for a short space of time to clear up all doubt.

The microscope is the safest and most reliable of all known means of diagnosis, and should always be resorted to in skillful hands when there is the least doubt.

Prognosis in the simple chancroid is favorable, but in the phagedenic, and when complicating some other disease, is always grave.

To attempt to go further into this subject would render this paper too long and tiresome, so I will hasten to a consideration of a few cases:

Case 1. Mrs. E. Married; respectable. Seen at Atlanta Polyclinic. Several months previous had become infected by husband; had been under treatment for some time previous to coming to clinic. (She informed me that her physician had used a strong caustic to the parts, but she did not improve.) On examination we found two ulcers—one on cervix close to os uteri and extending downward to vaginal mucous membrane for about three-quarters of an inch. The os had been almost entirely obliterated either by the application of the caustic mentioned above or the cicatrization following the ulcer. Another ulcer was situated on posterior vaginal wall and extending outward for about an inch. Pure carbolic acid was applied to these, but it seemed to aggravate them. The use of antiseptic washes, followed by insufflation of boric acid, caused a rapid cure, and the os was then operated on by Dr. Kime.

Case 2. Miss E., age 18 years—prostitute. This case came under my observation when a student. Shé had chancroidal ulcer of labia extending for short distance into vagina. I only had the use of a bivalve speculum and must not have been as careful in cleaning the parts as I should have been, for when she came the second time I found another ulcer higher up in vagina. This inoculation, which I attributed to the speculum, occurred several times. She was finally relieved by the use of pure carbolic acid, and on the external ulcer nitric acid followed by liberal use of iodoform; no other powder acted as well in this case, although I used many.

Before taking up the treatment I would like to quote some correspondence from several distinguished men on this subject.

Dr. William Goodell, of Philadelphia, says: "I have never seen a chancroid of womb proper, and have met with only one of labia majora."

Dr. H. C. Coe, of New York: "I rarely see a chancroid nowadays, possibly because I do little dispensary work and the class of patients whom I see at the Woman's Hospital are better than the average hospital cases. My partner, Dr. Jarmon, whose experience is large, assures me that (like myself) he does not believe that a chancroid can originate *de novo*. We have both seen chancroid in the lower fourth of the vagina, but *never* in the *body* of the uterus. Their occurrence in the cervix has been frequently described and figured by French authors, but they are exceedingly rare in our largest clinics here."

### TREATMENT.

When chancroids are pressing on the external genitals, and we have reasons to suspect their existence in vagina or on cervix, we should be very certain to thoroughly cleanse the external ulcers by the use of a strong solution of bichloride of mercury, followed by the application of pure carbolic acid before making a further investigation, else we will simply carry the chancroidal pus into vagina and inoculate a point higher up. Cleanliness is the *sine qua non* in the successful treatment of chancroids, keeping the secretions from the ulcer thoroughly washed away and neutralized by some good antiseptic; cleansing twice daily with hot antiseptic douches of bichloride of mercury 1-3000 or carbolic acid 5 per cent. These are best followed by the application of carbolic acid, C. P., and when the ulcers are small and not situated too close to an important opening, as the meatus urinarius, or organ (the bladder) the use of a strong caustic, nitric acid, until all diseased tissue is destroyed, is advisable. Neutralize the acid with solution of potassium hydrate or bicarbonate of soda, then apply iodoform, aristol, acetanilid, boric acid or some other good antiseptic. Aristol is said to possess special effects as a local anesthetic. The use of peroxide hydrogen, while in some cases has acted well, in others has had but little effect.

Persistence in the cleansing and the *proper* use of caustics followed by dry powders is the key to the successful management of these cases. The use of ointments of any sort in these cases is productive of more harm than benefit, for they simply cause a retention of the irritating discharges from the ulcer, thereby greatly aggravating the condition.

I agree fully with Dr. Keyes when he says, "perhaps the best treatment for simple uncomplicated chancroid, when not destroyed by caustic, is to cover the entire surface with iodoform."

BIBLIOGRAPHY—International Journal of Surgery, Keyes' Genito-Urinary Diseases, with Syphilis; Thomas & Munde, Gyneclogy, *et al.*

